# Star finches *Neochmia ruficauda* have a visual preference for white dot patterns: a possible case of trypophilia

**DOI:** 10.1007/s10071-022-01609-5

**Published:** 2022-03-16

**Authors:** Ayumi Mizuno, Masayo Soma

**Affiliations:** 1 Biosystems Science Course, The Graduate School of Life Science, Hokkaido University, Sapporo, Hokkaido Japan; 2 Department of Biology, Faculty of Science, Hokkaido University, Kita 10 Nishi 8, Kita-ku, Sapporo, Hokkaido 060-0810 Japan

**Keywords:** Estrildid finch, Plumage dot pattern, Sensory bias, Sensory exploitation, Signal evolution, Visual attention

## Abstract

Many animals have polka dot patterns on their body surface, some of which are known to have signalling functions; however, their evolutionary origins remain unclear. Dot patterns can trigger a fear response (trypophobia) in humans and are known to function as aposematic signals in non-human animals, suggesting that dots may deserve attention for biological reasons. Interestingly in many birds, plumage dot patterns serve for social/sexual signalling. To understand their evolution, we have focused on the sensory bias hypothesis, which predicts the role of pre-existing sensory preference driven by natural selection in shaping signal design. Our previous phylogenetic comparative study supported the hypothesis and showed that diet-driven visual preference promoted the evolution of plumage patterns, as there was an evolutionary correlation between termite-eating (white roundish gregarious prey) and the presence of plumage dot patterns in species of the family Estrildidae. This suggests that these species possess an intrinsic preference for dots. To test this, we compared the responses of an Estrildid species with dot plumage pattern (star finch *Neochmia ruficauda*) towards simultaneously presented monochrome-printed white dot vs white stripe patterns under both food-deprived and -supplied conditions. Overall, star finches preferred dots to stripes. They showed foraging-like behaviours almost only toward dots when hungry and gazed at dots frequently even when food was available, suggesting both hunger-related and hunger-neutral dot preferences. These results are rather surprising, given how strongly the subjects were attracted to abstract dot patterns without organic structure, but provided good support for the sensory bias hypothesis.

**Supplementary Information:**

The online version contains supplementary material available at 10.1007/s10071-022-01609-5.

## Introduction

Trypophobia, a fear response towards images that contain a cluster of dots or holes, is shown by many people and is argued to be an adaptive response to potentially harmful visual stimuli (Cole and Wilkins 2013; Kupfer and Fessler 2018; Kupfer and Le 2018) but is not well understood from a biological perspective (Kupfer and Fessler 2018). However, at least in many non-human animal species, having or paying attention to dot patterns can be crucial for the fitness of individuals, as conspicuous dot patterns serve as either aposematic signals in interspecific interactions or mating/social signals in within-species communication. This means that dot patterns can evoke aversion or attraction depending on the context, and the species of the signaller and receiver of dot signals. For example, dot patterns covering the entire body are known to function as warning signals against predators in ladybugs *Coccinella septempunctata* (Průchová et al. 2014) or in poison frogs *Dendrobates pumilio* (Siddiqi et al. 2004; Darst et al. 2006; Maan and Cummings 2012), whereas polymorphic dot patterns also contribute to individual recognition and mate choice in poison frogs (Summers et al. 1999; Siddiqi et al. 2004; Reynolds and Fitzpatrick 2007; Maan and Cummings 2008, 2009; Crothers et al. 2011). However, the functions of dot patterns have been studied in limited species compared with the vast numbers and diversity of animal species displaying dot patterns, leaving their evolution unresolved. In particular, it remains unclear why dot patterns have evolved as attractive social signals.

In birds, plumage dot patterns are very common (Somveille et al. 2016), and some of them are known to function as sexual or social signals (Alatalo et al. 1992; Roulin 1999; Crowhurst et al. 2012; Zanollo et al. 2012; Soulsbury et al. 2016; Soma and Garamszegi 2018). Individual birds with more conspicuous dot patterns (e.g. larger number of dots, or higher reflectance dots) can gain higher mating success (barn owls *Tyto alba*, Roulin 1999) or social dominance (diamond firetails *Stagonopleura guttata*, Crowhurst et al. 2012), suggesting that dot patterns, like other ornamental traits, evolved as signals as they reflect the quality of individuals owing to the production costs (Zahavi 1975, 1977; Andersson 1986; Grafen 1990). However, the cost associated with plumage dots is puzzling. Dot patterns usually appear as either achromatic spots on melanin-based feathers or melanin spots on whitish feathers. White plumage is known to require some maintenance cost to avoid bacterial growth, parasites (Kose and Møller 1999; Ruiz‐de‐Castañeda et al. 2012) or abrasion (Griggio et al. 2011), while melanin pigmented plumage also incurs production and maintenance cost (McGraw et al. 2002; Galvan and Alonso-Alvarez 2008; Piault et al. 2012; Guindre-Parker and Love 2014; Roulin 2016). Therefore, these traits can reflect immune challenge (Hanssen et al. 2008), hormone levels (Moreno and López-Arrabé 2021) or diets of individuals (McGlothlin et al. 2007). Even so, it is not clear whether having dots on plumage is more costly than total white or black plumage. Presumably, white spots may save resources for pigment production (Prum et al. 1999), but that does not explain why colourless parts take a particular shape (e.g., circle) and the same could be said for melanin spots as well.

As an alternative to the condition dependence mechanisms, the sensory bias hypothesis (Ryan and Keddy-Hector 1992; Endler and Basolo 1998; Ryan 1998; Rodríguez and Snedden 2004; Fuller et al. 2005) could possibly explain the evolution of dot patterns as signals. This hypothesis attempts to explain why specific traits evolved as mating signals, by focusing on female sensory preferences shaped under natural selection (Ryan and Keddy-Hector 1992; Endler and Basolo 1998; Ryan 1998; Rodríguez and Snedden 2004; Fuller et al. 2005; Fuller and Endler 2018). If particular characteristics are detected easily and perceived clearly by females, they can more likely be used as mate choice criteria than other less detectable/perceivable traits (Endler 1992; Ryan 1998). As a consequence, male mating signals that match female sensory systems would attract females. For example, in water mites *Neumania papillator*, males send vibratory signals that mimic those from their prey species to solicit female hunting response, leading to successful spermatophore transfer (Proctor 1991, 1992). A similar scenario can explain the evolution of colour pattern of body surface in some fish, such as guppies *Poecilia reticulata* or several *Goodeinae* species, where males attract females by having colour patterns that resemble foods that they prefer (Rodd et al. 2002; Garcia and Ramirez 2005). However, in birds, the idea that sexual ornament traits evolved from diet-driven preferences has not been well tested except for a few reports on bower decoration of bowerbirds (Madden and Tanner 2003; Borgia and Keagy 2006).

The sensory bias hypothesis yields the following three main predictions. First, signal features can be explained in the light of sensory system properties of signal receivers, i.e., females (e.g. Ryan and Rand 1993; Endler and Basolo 1998; Fuller and Endler 2018; Rosenthal 2018). Second, such signals can readily draw attention of females (e.g. Ryan and Cummings 2013). Finally, females should prefer to mate with males bearing such signal traits (e.g. Basolo 1990a, b; Ryan and Rand 1990; Rodd et al. 2002; Garcia and Ramirez 2005). Although these predictions are usually applied to explain the evolution of male signalling traits via female mate choice, the same explanations could be possible for the evolution of social signals, or sexual signals that are used for mutual mate choice. Considering that males and females evolved to have similar sensory systems, both sexes may show similar sensory preference towards particular stimuli, which can facilitate the signal evolution in any social contexts, including dominance interaction or individual identification.

Our previous phylogenetic comparative study indicated the role of foraging-related visual preference in the evolution of plumage patterns shared between the sexes of Estrildid finches (family: Estrildidae) (Mizuno and Soma 2020). Males and females of many species of Estrildid finches are characterised by conspicuous white dot patterns with signalling functions (Crowhurst et al. 2012; Zanollo et al. 2012; Soma and Garamszegi 2018), which evolved in association with a diet with spotty appearance (Mizuno and Soma 2020). Specifically, the species that regularly consume whitish small round gregarious prey, such as termites and ant larvae or eggs (Goodwin 1982; Payne 2010), tend to have white dot patterns (Mizuno and Soma 2020). In addition, their white dot patterns often cover the flanks but not the whole body (Morris 1958; Somveille et al. 2016; Soma and Garamszegi 2018), meaning that dots can attract attention in close distance communication, especially when Estrildid finches perform bilateral courtship display perching side by side (Goodwin 1982; Payne 2010). In such species, males are known to fluff up and fully display their dotted plumage during courtship display (Goodwin 1982). Having a visual preference for white dots would be crucial for both signalling communication and foraging in Estrildid species with dotted patterns. This could be applied to both sexes, explaining the evolution of mutual ornamentation driven by diet sensory preference (Mizuno and Soma 2020). Consistent with this idea, in a species of Estrildid fiches, diamond firetails *Stagonopleura guttata*, the number of dots is associated with the physical condition (Zanollo et al. 2012), and social dominance in females (Crowhurst et al. 2012).

**Fig. 1:**
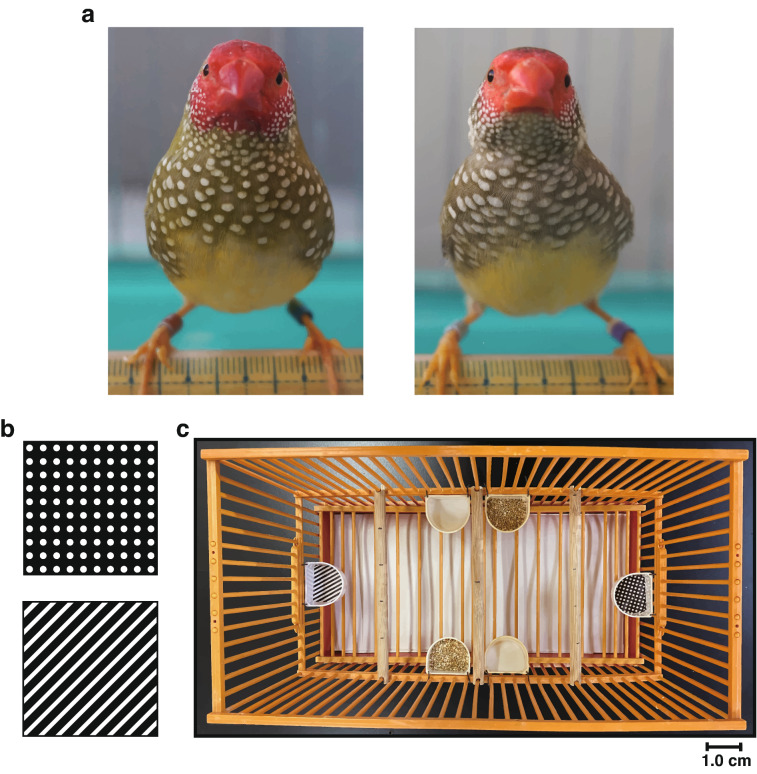
**a** Adult male (left) and female (right) star finch. **b** Dot and stripe stimuli. **c** Top view of the experimental cage, showing the food-supplied condition. The four cups in the middle contain water and seed mix, whereas the two on both sides contain stimulus-printed paper, which was replaced with plain white paper outside the tests

Therefore, we expected that the Estrildid species with white dot patterns would show strong visual attention to white dots (‘trypophilia’). To test this, we presented abstract dot patterns to the star finch *Neochmia ruficauda* (Fig. 1a), an Estrildid species with conspicuous white plumage dot patterns covering from face to flank. Visual attention to white dots can affect individual fitness in two ways. First, plumage dots in the species are highly likely to function as sexual and social signals (Crowhurst et al. 2012; Soulsbury et al. 2016; Soma and Garamszegi 2018), considering the sexual and among-individual variations in dot patterns. Second, images of dot patterns can indicate the presence of food sources, such as tiny seeds and termites (Goodwin 1982; Payne 2010). Therefore, we expect both hunger-related and hunger-neutral visual preferences for dot patterns in the star finch. In other words, hungry individuals would pay attention to dots, looking for foods, while non-starving individuals would also pay attention to dots because of their potential roles in social/sexual signalling. Considering the moderate sexual difference in the size of facial dot pattern areas (Goodwin 1982; see also Fig. 1a), we predict that males and females differ slightly in their responses to dot patterns. We prepared monochrome printed images of white dots and stripes as a matching stimulus and presented them simultaneously first under food-deprived conditions and then under food-supplied conditions to test their visual preference towards dots. If dot preference exists, the subject birds would show stronger responses towards dot stimuli than stripes. If such preference is driven by diet, the subject birds would show more visual attention towards dots in the food-deprived condition than in the following food-supplied condition. They would also show less or no foraging-like behaviours towards dots in the food-supplied condition after they had learned that dot stimuli are not food-rewarding in the preceding food-deprived condition. Alternatively, if dot preference is not solely for foraging, they may show visual attention towards dots even under food-supplied conditions, suggesting sensory bias towards dot patterns. These also mean that the idea of sensory bias should be rejected in unlikely case that dot preference exists only in the food-supplied condition.

## Methods

### Subjects

We used 15 male and 11 female adult star finches obtained from several local breeders. Each bird was identified with a unique combination of two-coloured leg rings. All birds were kept in unisex cages on a 12:12 h light:dark schedule (lights on 08:00–20:00) at approximately 25–26 °C and 50–60% humidity. They were provided with a finch seed mixture, cuttlebone, water, and fresh green vegetables every day, unless tested under food-deprived conditions. Each bird was tested in the experiments that were conducted between May and June 2019, or June and September 2020.

### Presented stimuli

We used monochrome dot—(φ 2.0 mm) or stripe—(W 2.0 mm) printed paper (Fig. 1b) as the experimental/control stimulus. We chose stripes as a control because they are widely seen on the plumage of other Estrildid species (Goodwin 1982; Payne 2010), and also because they are characterised by simple motif shapes comparable to dots. As the distances between motifs were set to 2.0 mm for both dot and stripe patterns, black areas were not exactly the same between the two stimuli (dot: 873.8 mm^2^; stripe: 668.8 mm^2^). The dot size was adjusted to match the largest white dots of Estrildid finches’ plumage patterns (Soma and Garamszegi 2018) to derive high responses from the birds. Stimuli were created using Adobe Illustrator CC 2018 (Adobe Systems, San Jose, CA), printed on white paper using an inkjet printer (DocuPrint C2110, FUJI XEROX, Tokyo, Japan), and set on the inner bottom of food cups (35.0 mm maximum diameter, 40.0 mm height) using double-sided tape. We used food cups to avoid neophobia and to prevent the stimuli from coming into the sight of birds easily without approaching.

### Experimental procedure

Each bird was individually introduced into an experimental cage (8.0 × 15.0 × 14.0 cm), which was equipped with food and water cups, and two other empty cups with white paper lining at the bottom, which was used for stimuli presentation during the tests (Fig. 1c). They were allowed to habituate there 1 day before the tests (Day 0) and tested for pattern preference under the food-deprived condition on Day 1 and food-supplied condition on Day 2. On Day 1, food cups were removed 3 h before presenting the stimuli and restored immediately after the experiment, whereas on Day 2, food was available all the time. Under each condition, we presented stimuli at the start of 1 h of behavioural recording using a video camera (GC-PX1, Victor, Tokyo, Japan), and removed them immediately after the end of the tests. The stimulus position was reversed from Day 1 to control for side preferences. The order of conditions was not randomised across birds.

### Behavioural quantification

To assess visual preference for each pattern, we measured the frequency of gazing and pecking behaviours towards each stimulus during each condition, assuming that gazing reflects visual preference (Dawkins 2002; Endler and Mappes 2017), and pecking is associated with foraging (Martin 2007). In this study, gazing refers to a bird looking down the stimuli, which was defined based on the perched position (i.e. on the perch or the floor close to the stimulus, or the edge of the stimulus cup), body/head orientation towards the stimulus (i.e. anterior half of body/head facing the stimulus), and the bill angle (i.e. pointing below horizontal). Pecking was defined based on the up-down head movements towards the stimulus shown by the bird standing on the stimulus cup. Frequently, the birds repeated pecking behaviours in a row, where we counted the total number of up-down movements. As pecking always accompanied gazing, we counted one gazing per series of peckings (see Online Resource 1). All videos were scored by the same observer (A.M.).

### Statistical analysis

To investigate whether the proportion of subjects’ responses to each stimulus (dots vs. stripes) deviated from those expected by chance (0.5), we ran intercept-only generalized linear mixed effect models (GLMM) with a binomial error distribution using the glmer function from the lme4 package (Bates et al. 2015). In these models, gazing/pecking frequency towards the two stimuli (dots vs. stripes) was entered as a bound response variable. We also tested the effect of experimental conditions (food-supplied vs. food-deprived) on dot preference using a GLMM with Poisson error distribution, with conditions specified as an independent variable. Possible sexual differences in response were also analysed using a separate GLMM in which sex was entered as an independent variable. The identity of the subject was incorporated in all three models as a random effect to address the possibility that individuals differed in their performance. All analyses were performed using R version 3.5.1 (R Core Team 2019).

## Results

**Fig. 2:**
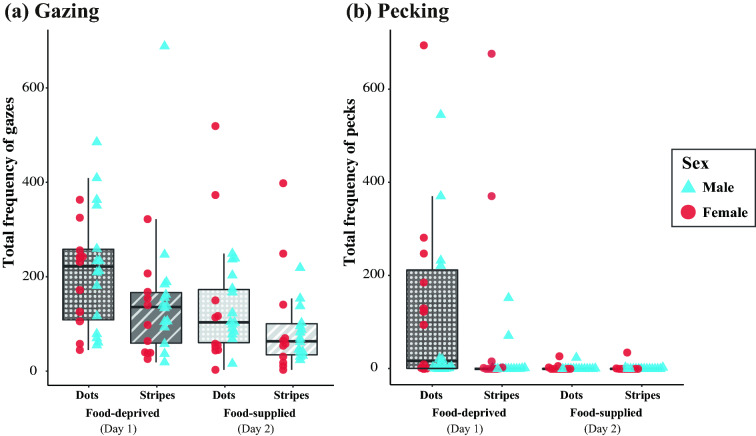
**a** Total frequency of gazes toward dot and stripe stimuli under the food-deprived and food-supplied conditions. **b** Total frequency of pecks toward dots and stripes under the food-deprived and food-supplied conditions

Star finches gazed at the white dot pattern more frequently than the stripe pattern under both food-deprived and -supplied conditions (deprived condition: *N* = 26, *Z* = 3.959, *P* < 0.001, supplied condition: *N* = 26, *Z* = 2.258, *P* = 0.024; Fig. 2a). Gazing preference towards dots was more pronounced under the food-deprived condition (*N* = 26, *Z* = 20.300, *P* < 0.001; Fig. 2a).

The pecking frequency (Fig. 2b) showed similar results with those for the gazing frequency (Fig. 2a), but with pronounced difference between the two (food-derived and -supplied) conditions. Star finches pecked white dot patterns more frequently than stripes under the food-deprived condition (*N* = 26, *Z* = 4.293, *P* < 0.001; Fig. 2b), but not under food-supplied conditions (*N* = 26, *Z* = 0.531, *P* = 0.596; Fig. 2b). The subject birds showed almost no pecking when food was supplied (Fig. 2b); therefore, the condition effect was statistically significant (*N* = 26, Z = 27.508, *P* < 0.001, Fig. 2b).

There were no sex differences in either gazing or pecking behaviour (gazing, food-deprived, *N* = 26, *Z* = − 0.958, *P* = 0.338, food-supplied, *N* = 26, *Z* = 0.254, *P* = 0.800, pecking, food-deprived, *N* = 26, *Z* = 0.282, *P* = 0.778, food-supplied, *N* = 26, *Z* = 0.527, *P* = 0.598).

## Discussion

Star finches showed both hunger-driven and hunger-neutral preference for dots by their frequent gazing and pecking at dot patterns compared with stripes, regardless of whether food was supplied (Fig. 2; Online Resource 1 and 2). This result is in accordance with our prediction but may seem rather surprising, given how strongly the subjects were attracted by completely abstract patterns without organic texture. Considering the visual acuity and depth perception of birds (Bischof 1988; Martin 2017; Caves et al. 2018), it is unlikely that they failed to perceive the clear image of dot patterns at a close distance, which suggests that they have an intrinsic visual preference for abstract dot patterns. If we could have controlled for prior visual experience of the subject birds (e.g., exposure to cage fences, round seed diet, or conspecifics plumage), that could give more support to the idea that star finches have the sensory bias for white dots.

Star finches’ visual preference for abstract dots can be partially explained in association with foraging. Hunger experience solicited foraging-like behaviour (i.e. pecking) towards dots (Fig. 2b), which was likely promoted by the method of stimuli presentation using food cups. However, their frequent gazing towards dots was likely not merely because of hunger, as they looked at dot patterns very frequently even under the food-supplied condition, after having experienced that the exact dot pattern was not food-rewarding the day before. These results suggest that dot stimuli for the star finch are worth paying attention to both in foraging and non-foraging contexts.

Evidence from previous research indirectly supports the idea that dots can play a role in within-species communication, i.e. sexual/social signalling (Alatalo et al. 1992; Roulin 1999; Summers et al. 1999; Siddiqi et al. 2004; Reynolds and Fitzpatrick 2007; Maan and Cummings 2008, 2009; Crothers et al. 2011; Crowhurst et al. 2012; Zanollo et al. 2012; Soulsbury et al. 2016; Soma and Garamszegi 2018). Like many Estrildid or other species that are characterised by dotted plumage patterns functioning for within-species signalling (Roulin 1999; Crowhurst et al. 2012; Zanollo et al. 2012; Soulsbury et al. 2016; Soma and Garamszegi 2018), star finches also bear conspicuous white dots covering from face to flank, wherein their visual attention to white dots would facilitate identification of conspecifics or potential mates. Although we did not find any sex difference in dot preference, this could be associated with the fact that both sexes have dot patterns. It is also possible that the sample size of this study was not sufficient to detect it. At least what is clear from the present results is that male star finches pay attention to dots like females do. This could be either because dot plumage pattern plays a role in social dominance in each sex (e.g. Crowhurst et al. 2012), or because dots function in mutual mate choice, given that most Estrildid finches are characterised by behavioural and morphological sexual signals shared between the sexes and functioning for mutual courtship (Gahr and Güttinger 1986; Geberzahn and Gahr 2011; Ota et al. 2015; Soma and Garamszegi 2015; Gomes et al. 2017; Soma and Iwama 2017; Soma 2018; Soma and Garamszegi 2018). It should be also noted that plumage patterns in Estrildid finches can have dual roles as sexual and social signals (Swaddle and Cuthill 1994; Crowhurst et al. 2012; Zanollo et al. 2013; Marques et al. 2016; Soma and Garamszegi 2018), making it hard to disentangle them. A possible direction for future studies is to examine the dot preference of species with clear sexual dichromatism in plumage dots, such as the zebra finch.

Lastly, but most importantly, the findings of this behavioural study, together with our previous phylogenetic comparative study (Mizuno and Soma 2020), have an important implication for how signalling traits originate and evolve in animals. According to the sensory bias hypothesis, sensory systems tuned for particular stimuli precedes the evolution of mating signals (Ryan et al. 1990; Ryan and Keddy-Hector 1992; Basolo 1995; Endler and Basolo 1998; Ryan 1998; but see also Ron 2008). This idea is supported by some research findings that females of closely related species with or without a mating signal show similar preferences, meaning that their common ancestors likely already had such a sensory preference, possibly because of diet (Proctor 1992; Garcia and Ramirez 2005). However, it should be noted that our previous and present studies did not directly examine whether plumage dots evolved as a result of sensory bias driven by foraging preferences. Theoretically, intrinsic dot preference could have originated from either plumage signalling or diet. We cannot completely deny the possibility that dietary choices arose as a result of an underlying preference for plumage dot pattern, but think it unlikely. It is because Estrildid’s common ancestors are assumed to lack white dot plumage patterns (Mizuno and Soma 2020), while all living Estrildid species are granivorous. For better understanding, it would be of great importance to apply interspecific comparative approaches to visual preference tests in the future. In addition, testing different colour combinations using the present test protocols might contribute to shedding light on how pattern signals are perceived and evolved in animals. What previous research overlooked is whether males share similar sensory preferences with females. As we have shown here, visual preference for particular stimuli may not be female-specific and can be a factor in the evolution of signalling traits that are shared between the sexes.

## Supplementary Information

Below is the link to the electronic supplementary material.

Supplementary file1 (MP4 152479 KB)

Supplementary file2 (MP4 95563 KB)

Supplementary file3 (XLSX 11 KB)

## Data Availability

Row data are provided as electronic supplementary material.

## Code availability

R code for the study can be provided on request.
